# Effect of adiposity on tissue-specific adiponectin secretion

**DOI:** 10.1371/journal.pone.0198889

**Published:** 2018-06-20

**Authors:** James Reneau, Matthew Goldblatt, Jon Gould, Tammy Kindel, Andrew Kastenmeier, Rana Higgins, L. Rosemary Rengel, Katherine Schoyer, Roland James, Brittaney Obi, Andrea Moosreiner, Kay Nicholson, Daisy Sahoo, Srividya Kidambi

**Affiliations:** 1 Department of Medicine, Medical College of Wisconsin, Milwaukee, Wisconsin, United States of America; 2 Department of Surgery, Medical College of Wisconsin, Milwaukee, Wisconsin, United States of America; 3 Department of Obstetrics and Gynecology, Medical College of Wisconsin, Milwaukee, Wisconsin, United States of America; 4 Clinical and Translational Science Institute, Medical College of Wisconsin, Milwaukee, Wisconsin, United States of America; John Hopkins University School of Medicine, UNITED STATES

## Abstract

Circulating adiponectin levels are lower in individuals with increased BMI and central adiposity. However, they are paradoxically higher in those with peripheral adiposity. We hypothesized that adiponectin secretion from central and peripheral adipose tissue depots may be associated with adiposity levels and its distribution. A total of 55 subjects (69% women) undergoing elective abdominal surgery (mean age: 53 ± 13 years) were recruited. Health history, anthropometrics, and cardiovascular disease risk factor measurements were obtained. Subcutaneous adipose tissue (SAT) and visceral adipose tissue (VAT) samples were obtained and cultured. Media was collected after 24hr and adiponectin released into the medium was measured using ELISA. We found that mean adiponectin levels from SAT and VAT in all subjects were 17.14±15.27 vs. 15.21±14.28 pg/ml/mg of tissue respectively (p = ns). However, adiponectin secretion from VAT correlated negatively with BMI (r = -0.31, p = 0.01), whereas there was no relationship with SAT (r = 0.08 p = 0.61). Similarly, waist circumference and estimated VAT percentage were both negatively correlated with VAT secretion of adiponectin (r = -0.35, p = 0.01 and r = -0.36, p = 0.02 respectively). These negative correlations were significant only in women on gender-stratified analyses. Adiponectin secretion from VAT decreases with increases in adiposity, while SAT secretion remains unchanged, especially in women. This observation may explain lower circulating adiponectin levels in individuals with central obesity. Further studies are needed to explore the mechanism behind this discrepant adiponectin secretion from SAT and VAT with increases in BMI, particularly among women.

## Introduction

Central adiposity with visceral adipose tissue (VAT) accumulation is a risk factor for the development of type 2 diabetes and cardiovascular diseases [1]. In contrast, excessive adipose tissue (AT) accumulation in peripheral subcutaneous adipose tissue (SAT) depots does not carry the same metabolic disease risk and, in fact, may afford some degree of protection against the same [2, 3]. In addition to AT’s function as an energy storage and insulating organ, it is also a known source of large number of secretions (adipokines) that include hormones, pro- and anti-inflammatory markers, and other bioactive substances [4]. These secretions differ between various AT depots and may explain the differential metabolic effects of each depot’s abundance [5].

Adiponectin is one such adipokine, with insulin-sensitizing, anti-inflammatory, and anti-apoptotic properties, that is primarily secreted by AT in adult humans [6]. Lower adiponectin levels are associated with type 2 diabetes and cardiovascular disease risk and higher levels of the same are associated with protection from cardiometabolic diseases [7, 8]. Even though AT is the major source of adiponectin *and* adiponectin is the most abundant adipose-specific protein secreted in the human body [9], its levels decrease with increases in adiposity, especially central adiposity [10].

Adding to the intrigue, certain obese individuals who are metabolically healthy were noted to have paradoxical hyperadiponectinemia [11]. We and others have recently shown that these obese individuals with paradoxical hyperadiponectinemia have greater lower body adiposity (i.e. higher peripheral adiposity rather than central or visceral adiposity) compared to obese individuals with lower adiponectin levels [12]. We also showed that higher abdominal SAT-to-VAT ratios are associated with increased circulating adiponectin levels, suggesting differential secretion of adiponectin from different AT depots [13]. We, therefore, hypothesized that adiponectin secretion from VAT and SAT depots may be associated with adiposity levels and its distribution.

## Methods

### Subjects

Fifty-five subjects undergoing elective abdominal surgeries were recruited from Froedtert Hospital and the Medical College of Wisconsin, Milwaukee, WI. All subjects underwent pre-operative phenotyping during the time-period between initial consultation and their actual surgery date (typically 4 weeks). Forty percent of the subjects underwent bariatric surgery. None of the subjects who underwent bariatric surgery were placed on a pre-operative high-protein liquid diet, however, were advised to follow a 1200 kilocalorie diet for at least two-weeks prior to surgery. Remaining surgeries included hernia repair (50%), cholecystectomy (2%), laparoscopic fundoplication (4%), and ovarian cystectomy (4%). This study was approved by the Froedtert Hospital and the Medical College of Wisconsin’s Institutional Review Board. All participants provided a written informed consent. All methods were performed in accordance to the IRB protocol and relevant guidelines and regulations.

### Phenotyping

Standardized anthropometric measurements were performed by a registered dietitian following the Center for Disease Control and Prevention’s National Health and Nutrition Examination Survey (NHANES) Anthropometry Procedures Manual [14]. Height and weight were obtained to calculate body mass index (BMI). Waist, hip, and thigh circumference measurements were taken directly on the skin with a Gulick II Tape Measure (Country Technology, Inc., Gay Mills, WI). Waist circumference was measured at the level of the umbilicus. Hip circumference was measured over the maximum extension of the buttock with participant’s feet placed together. Whole-body composition including total fat mass, lean body mass, and visceral fat percentage were measured using a dual-energy X-ray absorptiometry (DXA) using an iDXA scanner (GE Lunar Medical Systems, Madison, Wisconsin).

### Laboratory measurements

C-reactive protein was measured using an enzyme-linked immunosorbent assay (ELISA) (MP Biomedicals, Santa Ana, CA) with sensitivity and coefficient of variation (CV) at 2.81 ng/ml and <9.2% respectively. Total and high molecular weight adiponectin were analyzed using ELISAs (R&D systems, Inc. Minneapolis, MN) with sensitivity and CV at 0.79 ng/ml and <6.9% and 0.195 ng/ml and <8.6% respectively. Free fatty acids were measured using enzyme-based methods and quantified by colorimetric assays (Wako Diagnostics, Richmond, VA) with sensitivity of 0.0014 mEq/L and CV <2.7%. Sandwich ELISA kits (R&D Systems, Inc. Minneapolis, MN) were used to measure interleukin -6, interleukin-10, and tumor necrosis factor -alpha (TNF-α) with sensitivity and CV of 0.70 pg/ml and <6.4%, 3.9 pg/ml and <7.5% and 1.6 pg/ml and <5.2% respectively. Leptin, was also analyzed using ELISA (R&D Systems, Inc. Minneapolis, MN) with sensitivity and CV at 7.8 pg/ml and <5.4% respectively.

### AT sampling

Paired VAT and SAT samples were collected at the time of surgery and were immediately transported to the laboratory in Hank’s medium. Samples were taken from the omental fat depot adjacent to the greater curvature of the stomach during laparoscopic surgery using endoscopic scissors. No cautery was used and samples were placed in Hank’s medium and transported to the laboratory within 30–45 minutes of collection.

### AT culture

Once the tissue was received by the laboratory, it was weighed after removing as much liquid as possible. All steps were performed in the culture hood with sterile equipment by the same technician. Tissue was minced into smaller fragments to prevent hypoxia and improve adipokine secretion. Tissue was then placed into 10 cm culture dishes containing 10 mL Dulbecco’s Modified Eagle’s Medium with GlutaMax and high glucose (ThermoFisher Scientific, Waltham, MA) supplemented with 10% fetal bovine serum. Tissue was incubated at 37°C with 5% CO_2_. Media was changed at 2 hours to remove lipid droplets. Media was collected after 24 hours, centrifuged at 1000 g for 15 minutes and stored in -80°C until ready for measurements. Medium was also collected after 4 hours in 15 subjects (27%). Adiponectin levels released into the medium were initially measured using Bioplex methods, and further confirmed by an adiponectin ELISA. All fifty-five subjects had at least one tissue-specific adiponectin secretion level. Of which, 50 subjects had VAT adiponectin secretion values and 46 subjects had SAT secretion levels. Both values were present on 41 subjects.

### Immunoblot analyses

Presence of adiponectin protein in AT was confirmed by immunoblot analyses. Whole tissue, in the presence of protease inhibitors, was sonicated 5 times (3 second bursts each), with cooling periods on ice between sonications. Tissue was placed at -80 °C for 20 minutes, centrifuged at 12,000 rpm for 15 min at 4 °C. Lysate from the lower layer was collected and protein levels were determined by the Lowry method. Lysates (50 μg protein) were separated by 15% SDS-PAGE. Proteins were transferred to nitrocellulose membranes, and adiponectin was detected using a primary adiponectin monoclonal antibody (Thermo 19F1). Beta-actin was detected as a loading control.

### Statistical analysis

All the subjects were consecutively recruited. Descriptive statistics (mean, standard deviation, sample proportions etc.) were used to describe the distribution of disease states, measures of adiposity, tissue adiponectin levels, circulating adipokines, and other demographic/clinical/laboratory characteristics within the study sample. All data are presented as the mean ± standard deviation. Correlation coefficients and linear regression analyses were used to describe the relationship of tissue adiponectin with adiposity measures. Two-sample t-tests were used to test for differences across groups.

## Results

A total of 55 subjects were recruited of which 69% were women. Demographics, anthropometrics, and metabolic parameters of all subjects are shown in Table 1. The range of BMIs included in this group was 19–62.4 kg/m^2^. Nine subjects (16%) were non-obese (BMI <30 kg/m^2^). Twenty-one subjects (39%) had BMI over 40 kg/m^2^. Women had higher total body fat percentages and leptin levels compared to men; however, the percentage of VAT was similar to men. In the overall sample, BMI correlated negatively with total and high molecular weight adiponectin levels (r = -0.29, p = 0.03 and r = -0.26, p = 0.07 respectively) and positively with C-reactive protein (r = 0.52, p = 0.0001), interleukin-6 (r = 0.39, p = 0.005), and leptin (r = 0.49, p = 0.0002). When men and women were analyzed separately, the negative relationship between BMI and circulating adiponectin level was stronger among men compared to women (r = -0.61, p<0.05 vs. r = -0.15, p = ns). Additional correlations are shown in Table 2.

**Table 1:** Baseline demographic, anthropometric, and metabolic characteristics (mean ± SD).

Characteristics	All subjects (n = 55)	Women (n = 38)	Men (n = 17)
Age (years)	53 ± 13	53 ± 12	53 ± 14
BMI (kg/m^2^)	41 ± 11	42 ± 11	38 ± 9
Waist circumference (cm)	123 ± 20	122 ± 21	123 ± 19
Hip circumference (cm)	132 ± 22	134 ± 22	123 ± 25
Thigh circumference (cm)	64 ± 9	65 ± 9	63 ± 10
Waist-to-Hip Ratio	0.96 ± 0.16	0.92 ± 0.09	1.04 ± 0.25 *
Total body fat mass (%)	39 ± 8	42 ± 6	33 ± 8 ***
Total fat mass (kg)	54 ± 21	57 ± 20	48 ± 21
TFM adjusted for BMI	1.27± 0.25	1.28 ± 0.22	1.23 ± 0.32
Lean body mass (kg)	58 ± 13	53 ± 10	69 ± 11***
LBM adjusted for BMI	1.45 ± 0.36	1.26 ± 0.21	1.87 ± 0.24***
Estimated visceral fat mass (%)	39 ± 11	38 ± 10	42 ± 11
Type 2 diabetes (%)	40	45	29
Hypertension (%)	53	58	41
Bariatric surgery (%)	40	45	29
Total adiponectin (mcg/ml)	6.91 ± 4.22	7.30 ± 3.97	6.04 ± 4.77
HMW adiponectin (mcg/ml)	4.05 ± 3.79	4.19 ± 3.47	3.47 ± 4.52
Free fatty acids (mmol/l)	0.60 ± 0.28	0.64 ± 0.28	0.49 ± 0.27
C-reactive protein (nmol/l)	67.5 ± 63.9	72.5 ± 69.0	56.7 ± 51.3
IL-6 (pg/ml)	4.27 ± 3.38	4.71 ± 3.82	3.26 ± 1.75
IL-10 (pg/ml)	7.47 ± 9.23	5.94 ± 6.90	7.56 ± 12.30
TNF-alpha (pg/ml)	7.78 ± 5.99	7.16 ± 5.75	9.11 ± 6.39
Leptin (ng/ml)	45.51 ± 35.43	55.12 ± 38.32	23.89 ± 11.07**

SD: Standard deviation BMI: Body mass index TFM: Total fat mass LBM: Lean body mass

HMW: High molecular weight IL: Interleukin TNF: Tumor necrosis factor

p-values: * <0.05 ** <0.01 *** <0.001

**Table 2:** Correlations of BMI with metabolic characteristics.

Metabolic parameters	Overall	Females	Males
HMW Adiponectin	-0.26	-0.15	-0.61*
Free fatty acids	0.18	0.25	-0.13
C-reactive protein	0.52***	0.54***	0.37
IL-6	0.39**	0.42*	0.10
IL-10	0.20	0.12	0.42
TNF-alpha	0.06	-0.03	0.44
Leptin	0.49***	0.50***	0.59*

HMW: High molecular weight IL: Interleukin TNF: Tumor necrosis factor

P-values: * <0.05 ** <0.01 *** <0.001

**Fig 1:**
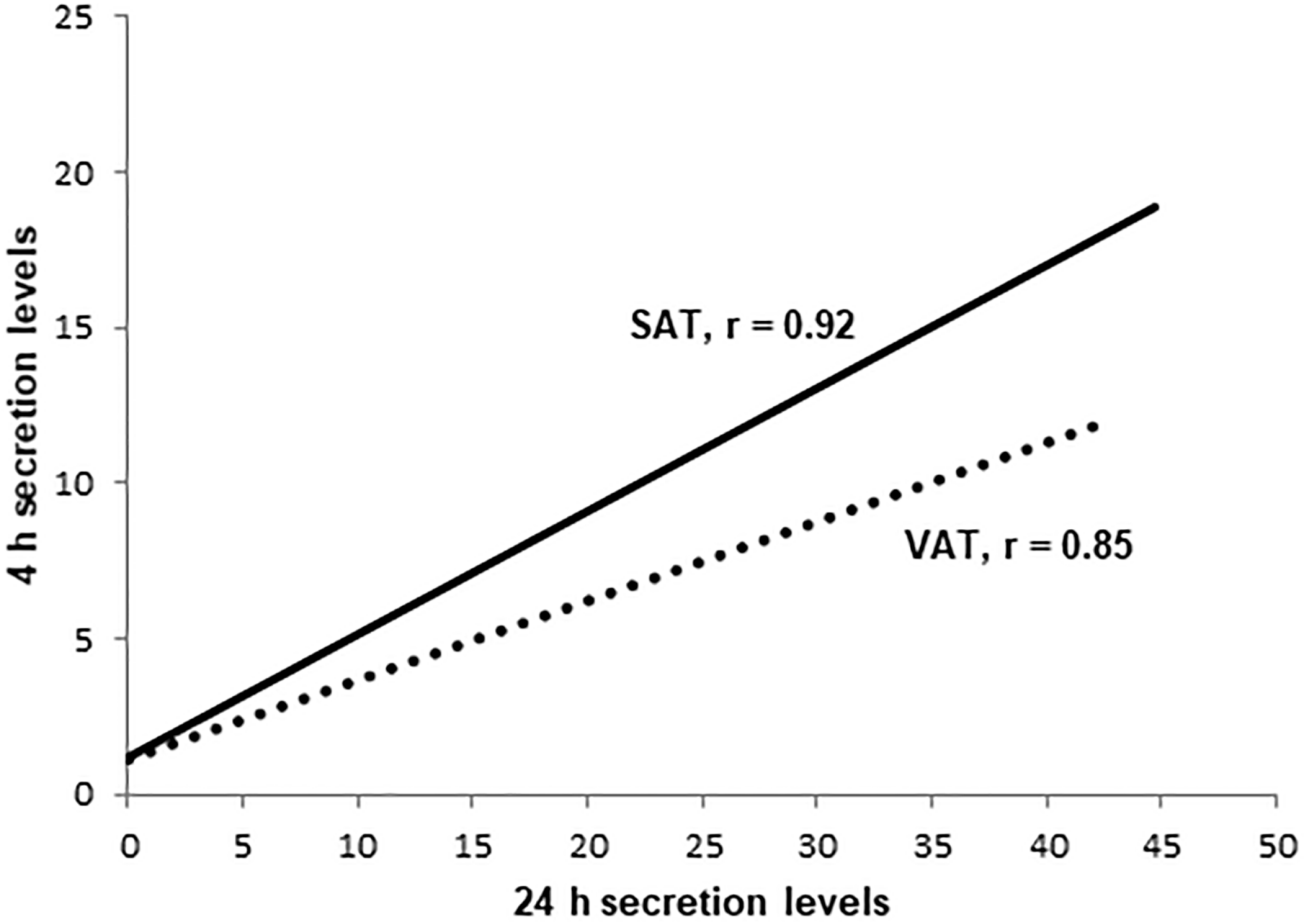
Secretion levels of adiponectin from both visceral adipose tissue (VAT) and subcutaneous adipose tissue (SAT) at 4 hours and 24 hours were highly correlated with each other (p < 0.001).

**Fig 2:**
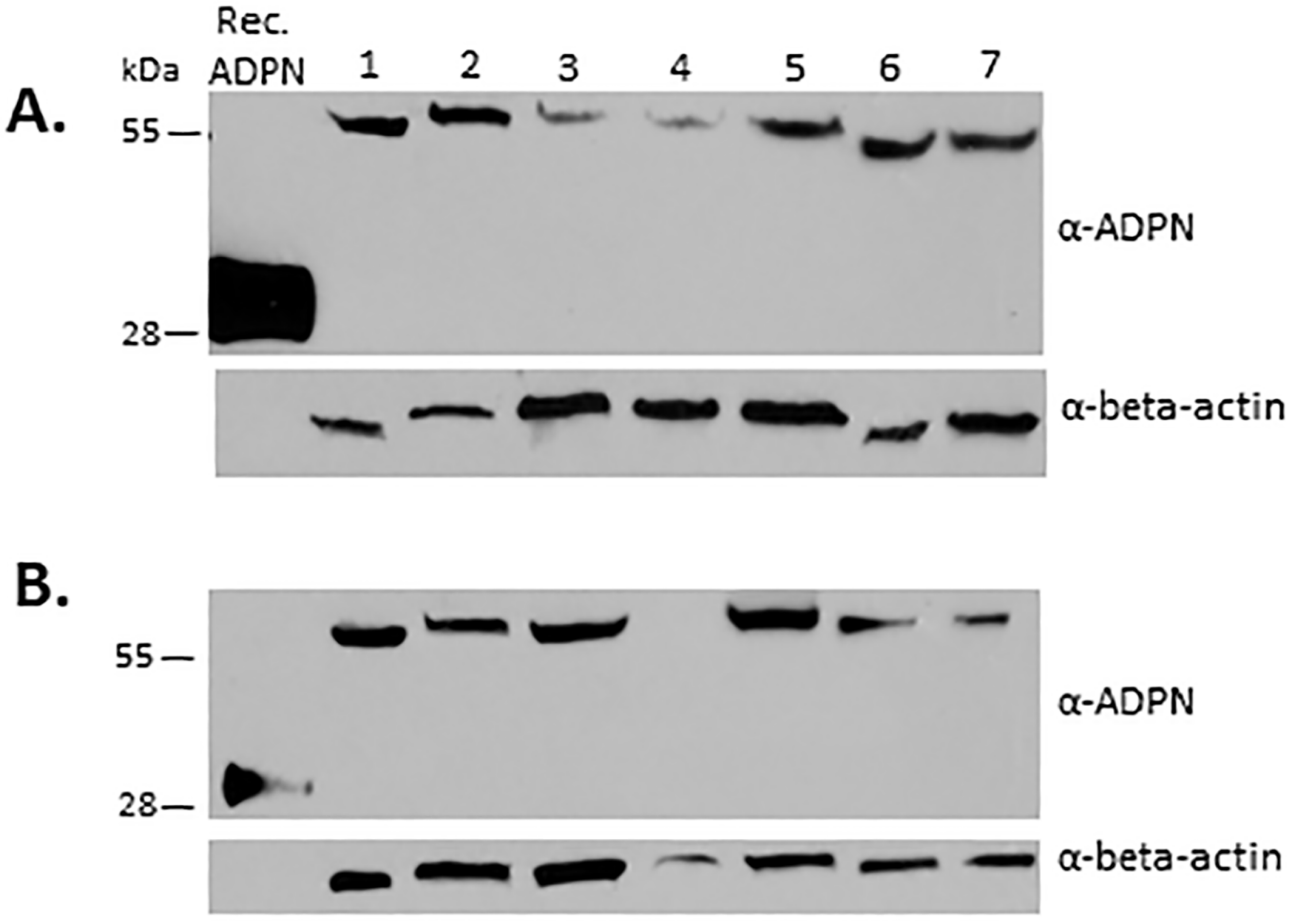
Visceral adipose tissue (VAT) (A) and subcutaneous adipose tissue (SAT) (B) tissue homogenates (50 μg protein) were separated by 15% SDS-PAGE and transferred to nitrocellulose membranes for immunoblot analyses. Adiponectin (ADPN) levels from paired SAT and VAT samples are shown for 7 different subjects. Beta-actin loading control and monomeric recombinant adiponectin (~30 kDa) are shown).

Depot-specific adiponectin secretion levels into the media were initially measured by Bioplex and further confirmed by ELISA. All results presented are from ELISAs which were deemed more reliable between the two assays. However, depot-specific adiponectin levels detected using both methods were highly correlated (p<0.0001). We further evaluated depot-specific secretion adiponectin into the media at both 4 hours and 24 hours (n = 15 subjects). We found that secretion levels at 24 hours were significantly higher than secretion levels at 4 hours in both SAT (mean ± SD: 15.39 ± 13.67 vs. 7.38 ± 5.79 pg/mL/mg of tissue, p = 0.04) and VAT (13.43 ± 13.71 vs. 4.63 ± 4.02 pg/mL/mg of tissue, p = 0.03), hence we chose to limit our measurements to 24-hour time points in subsequent subjects. However, there were significant correlations between levels measured at 4 hours and 24 hours from both SAT (r = 0.92, p < 0.0001) and VAT (r = 0.85, p = 0.0001) (Fig 1). We also measured secretion levels at 48 and 72 hours in 10 initial subjects, and these levels were significantly lower than 24-hour levels (almost negligible in some cases). We, therefore, do not present 48 and 72-hour results. We further confirmed the presence of adiponectin protein in paired SAT and VAT samples by immunoblot analysis (Fig 2).

In the overall sample, mean secretion levels from SAT and VAT were 17.14 ± 15.27 (interquartile range: 23.68) vs. 15.21 ± 14.28 (interquartile range: 20.20) pg/mL/mg of tissue (mean ± SD) at 24 hours. These levels were not significantly different from each other in the overall sample (p = 0.52). In subjects with BMI ≤ 40 kg/m^2^, SAT and VAT secretion of adiponectin levels were 16.40 ± 13.13 vs. 19.07 ± 16.65 pg/mL/mg of tissue respectively (p = 0.54). However, in subjects with BMI over 40 kg/m^2^, VAT secretion of adiponectin trended lower compared to SAT (17.85 ± 17.33 vs. 12.52 ± 11.00 pg/mL/mg of tissue, p = 0.07).

**Fig 3:**
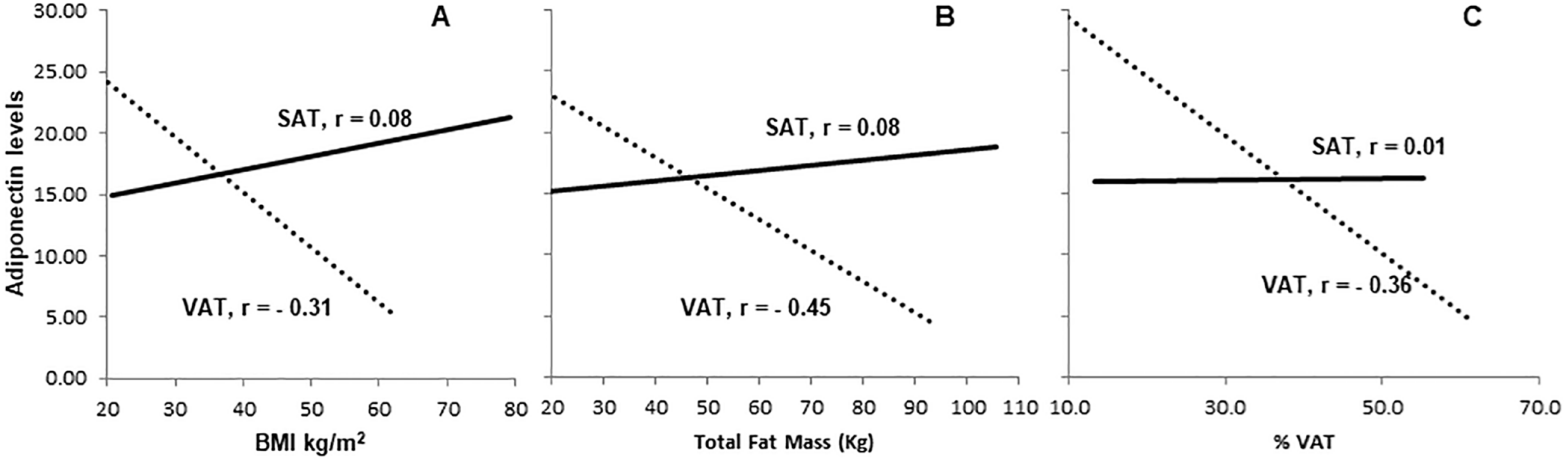
A: Secretion levels of adiponectin from visceral adipose tissue (VAT) is negatively associated with BMI while there was no association with subcutaneous adipose tissue (SAT) (p < 0.05) B: Secretion levels of adiponectin from VAT is negatively associated with total fat mass while there was no association with SAT (p < 0.001) C: Secretion levels of adiponectin from VAT is negatively associated with visceral fat percentage while there was no association with SAT (p < 0.05).

In the overall sample, SAT secretion of adiponectin was not correlated with BMI (r = 0.08, p = 0.61), while VAT secretion of adiponectin was negatively correlated (r = -0.31, p = 0.03) (Fig 3). In addition, total fat mass adjusted for BMI was negatively correlated with VAT secretion of adiponectin (r = -0.45, p = 0.001). Similarly, waist circumference and estimated VAT percentage were both negatively correlated with VAT secretion of adiponectin (r = -0.35, p = 0.01 and r = -0.36, p = 0.02 respectively). SAT secretion of adiponectin was not significantly associated with any of these adiposity measures.

Gender-specific associations of various adiposity measures with depot-specific adiponectin secretion are shown in Table 3. The association between BMI and VAT secretion of adiponectin differed between men (r = 0.06, p = ns) and women (r = -0.40, p = 0.02). In addition, no statistically significant correlation was noted between BMI and SAT secretion of adiponectin among both men (r = 0.27, p = ns) and women (r = 0.03, p = ns). All ‘r’ values in women showed moderate association (0.4–0.55), and the association of adiponectin with waist circumference and total body adiposity reached statistical significance even after adjusting for multiple comparisons (p<0.006), while the same associations in men were not significant (Table 3).

**Table 3:** Associations of adiposity measures with tissue specific adiponectin secretion.

	SAT adiponectin secretion			VAT adiponectin secretion		
	Overall	Women	Men	Overall	Women	Men
BMI	0.08	0.03	0.27	-0.31*	-0.40*	-0.06
Waist circumference	0.11	-0.09	0.43	-0.35*	-0.55***^¶^	0.24
Total fat mass/BMI	-0.03	-0.10	0.05	-0.45***	-0.57***^¶^	-0.28
Visceral fat percentage	0.01	-0.06	0.09	-0.36*	-0.41*	-0.27

BMI: Body mass index SAT: Subcutaneous adipose tissue VAT: Visceral adipose tissue

p-values: * ≤ 0.05 ** ≤ 0.01 *** ≤ 0.001

Corrected p-value for multiple comparisons for correlations among men and women: ^¶^ <0.006

## Discussion

In this study, we found that the secretion of adiponectin from VAT decreased with increases in BMI, total body fat, and VAT. We also found that the ability to secrete adiponectin from SAT is preserved with increases in these adiposity measures. In addition, these relationships were stronger in women compared to men. This observation may explain lower circulating adiponectin levels in individuals with central obesity. Relatively preserved secretion of adiponectin from SAT may also be responsible for the paradoxical hyperadiponectinemia noted in some metabolically healthy obese (MHO) individuals. These findings are in line with previous observations that AT secretory characteristics differ based on its location [15].

Adiponectin, secreted exclusively by mature adipocytes, is one of the largest products of AT [9]. Despite AT being a predominant source for adiponectin, numerous studies have shown that adiponectin levels are lower in individuals with obesity [16]. While it is not entirely understood why circulating adiponectin levels decrease with increased adiposity, it is hypothesized to be down-regulated at the level of gene expression in dysfunctional adipocytes present in obesity [17]. Adiponectin secretion has been shown to be inhibited by pro-inflammatory cytokines and oxidative stress frequently present in individuals with obesity [16, 18]. Circulating high molecular weight adiponectin levels showed negative trends with BMI in the current study as well.

Recent observations of higher than expected adiponectin levels in a sub-group of healthy obese subjects (paradoxical hyperadiponectinemia) have turned the discussion towards the role of adiposity distribution in determining adiponectin levels [12, 19]. We have previously shown that individuals with higher SAT have higher circulating adiponectin levels despite obesity [13]. These findings led us to hypothesize that AT depots differ in their ability to secrete adiponectin with increases in adiposity which we found to be accurate in this study. These findings are consistent with previous studies that evaluated differential secretion of adiponectin between AT depots [20]. In a 2004 study of 16 women, Fain et al. showed that VAT secretion of adiponectin was higher in women with an average BMI of 32 Kg/m^2^ in comparison to women with an average BMI of 42 Kg/m^2^ [21]. In another study of 52 women undergoing abdominal hysterectomies, adiponectin release by omental and subcutaneous adipocytes was similar in lean women while adiponectin release by omental adipocytes was significantly reduced in obese women [15]. At the same time, adiponectin release from subcutaneous cells was not associated with any measure of adiposity in obese women [15]. Similarly, a strong negative correlation was noted between BMI and adiponectin secretion from omental cells in a small study of 9 subjects, while secretion from subcutaneous adipocytes was unrelated to BMI [20]. In another study of 25 women undergoing elective gynecological surgeries, adiponectin expression was higher in SAT compared to omental AT in both lean and overweight women [22]. Furthermore, Chen and colleagues found that omental expression of adiponectin was much higher after weight loss with gastric bypass surgery when compared to age-matched obese controls undergoing the same surgery and matched non-obese controls [23]. Our study evaluated AT (in contrast to adipocytes) secretion of adiponectin in a relatively large sample size of both men and women (in contrast to studies limited to women). Due to the influence of surrounding stromal and inflammatory cells on adipocyte secretions, AT instead of isolated adipocytes was investigated in this study. In addition, we measured total and visceral adiposity by DXA scan along with BMI and BMI is not always a good representative of body composition [24]. We must acknowledge that there are important differences in the various methodologies used in these studies including the use of RT-PCR or Northern Blot techniques to measure mRNA levels or measurement of adiponectin release into the medium, a method we chose to use in our studies [20, 25, 26].

Even though the exact mechanisms behind decreased adiponectin secretion from VAT with increases in BMI have not been elucidated, we can postulate that differences in adipogenic potential, hormonal influences, and perhaps gut microbiota may play a role [26–31]. Further exploration of these factors may lead to understanding why VAT secretion of adiponectin decreases with obesity. Understanding these mechanisms will help us understand pathogenicity of central adiposity and potential to develop novel treatment options.

Significant gender differences have been noted in total plasma adiponectin levels in previous studies [32]. Adiponectin concentration is consistently lower in men than in women when adjusted for BMI and is considered to be due to sex hormones and adiposity distribution [33]. Circulating testosterone levels and testosterone supplementation have been associated with lower circulating adiponectin levels in in vivo studies of both men and rodents, though direct influence of sex hormones on adipocytes is less clear [34, 35]. Women have much higher quantities of SAT (gynoid obesity) compared to men and may also explain higher adiponectin levels especially in women with higher BMI [36, 37]. This sexual dimorphism seems to be not associated with differing concentrations of estrogens as studies did not show significant differences in adiponectin levels among pre- and post-menopausal women and women on hormone replacement therapy [38]. In addition, novel mechanisms involving secreted frizzled-related proteins (SFRPs) and VAT-derived serine protease inhibitor, serpinA12 (Vaspin) have been proposed [39]. Although it is unclear why increases in adiposity affect adiponectin secretion from VAT in women more adversely than men, it may explain greater detrimental consequences of central adiposity and metabolic syndrome in women [40, 41].

There are several strengths to our study including detailed phenotyping of subjects prior to obtaining AT and the fact that none of the bariatric surgery patients were on 2- week liquid diet prior to obtaining AT which may affect cellular processes due to acute weight loss prior to surgery. However, all bariatric surgery patients were asked to follow a low-calorie diet (though calories were not specified) and that may have affected the results as well. One other weakness of this study is the limited number of subjects which precluded stratified analyses by comorbidities and medication use. Approximately 70% of patients with type 2 diabetes were on treatment (50% on insulin and 50% on either sulphonylureas or metformin or a combination), however, we were unable to stratify them. We had fewer men compared to women. The population in our study had highly diverse BMI, and many of the subjects were obese with only a small group in the normal BMI category and it is quite possible that the gender differences we observed were simply due to limited sample size. We also acknowledge that since 40% of our study population has BMI > 40 Kg/m^2^, these results may not be applicable to an entirely normal weight or an overweight population. In addition, this is a cross-sectional study so we are unable to show any temporal association between adiponectin secretion changes and increases in BMI. We used DXA scan to measure adiposity and which is not as accurate as magnetic resonance imaging (MRI) for measuring fat depots. However, DXA scan was feasible in our population due to some subjects being too large to fit into an MR scanner. We also acknowledge that we did not assess the effect of clock gene expression on adiponectin secretion from these samples.

In summary, our results demonstrate differential secretion of adiponectin by VAT and SAT depots and may explain why circulating adiponectin levels are lower among those with central adiposity. Conversely, relatively preserved secretion of adiponectin from SAT may explain paradoxically higher adiponectin levels in MHO individuals. Since adiponectin is considered cardioprotective, elucidating the mechanisms behind these secretory patterns may help develop therapeutic strategies aimed at reversing molecular changes that lead to lower adiponectin secretion from VAT in centrally obese individuals.

## Supporting information

S1 Appendix Data and related metadata underlying the findings presented in this manuscript. (XLSX)
